# A Curious Statement

**Published:** 1884-08

**Authors:** 


### A CURIOUS STATEMENT.

J. T. Kent, M. D.: As you have known me and been my true friend for eight years, and as I think you have confidence in me to believe that I would not knowingly deceive you in one iota, I make the following statement to you as a physician, in strict confidence as to my personal identity, whatever use you may be pleased to make of this. An added affidavit could not make it any truer or qualify one word.

It has always been my sentiment that he who has others dependent upon him and backs out of life's ills by suicide is a coward and a sneak, and not necessarily insane. I hold that the suicide who in the act makes a mess of himself for others to clean up is more hog than gentleman. Of late years, having no one dependent upon me, and steadily failing in health, I have come to the conclusion that should the time ever come when I found myself useless, hopeless, penniless, dependent upon others and no prospect of improvement, perhaps the enforced companion of vile and ignorant paupers, that I had or would take the right to return to the dreamless sleep from which I was awakened and squeezed headlong into "this vain and wicked world," January 10th, 1841. Like Longfellow's Spanish student, I reasoned,

> "What is death?
> To leave all disappointment, care, and sorrow,
> To leave all falsehood, treachery, and unkindness;
> All ignominy, suffering, and despair,
> And be at rest forever! O dear heart!
> Be of good cheer; when thou shalt cease to beat,
> Then shalt thou cease to suffer and complain."

On the morning of February 22d, the condition above referred to seemed to have fully arrived; being sick, destitute, no friends, no aid, no hope, nothing but misery and despair ahead, I crawled out and bought a phial of Powers & Weightman's *Sulph. morphia*, sixty grains, and the phial was full. It was bought at a drug store where I traded. I saw it taken from an ounce package in stock, and was handed to me without question or remark.* To be sure of its quality, I tested it with the tongue and found the usual bitter taste, though I never had used that or any other drug at all. The remainder of the day I employed "setting the house in order," disposing of my few effects, writing a few messages to friends—all in a calm frame of mind, like one who approaches the end of a long and weary journey where soon he is to enjoy blessed rest. At 6.30 P. M. I shook about one-quarter the contents of the phial—not less than fifteen grains, probably more—into a glass, mixed well with water. I poured out a big horn of good whisky long had on hand, drank it, and drank the contents of the glass to the dregs. Then, lying down on the bed on my right side, I said, "Good-bye, vain, weary world," and fell asleep.

In this house there boards a lady and gentleman in whose family I had boarded for a year, and they esteem me highly. While long ill the kind-hearted lady had tended me with a sister's anxiety and care. Passing my room just before midnight, she saw that the gas was alight within. She knocked. No answer. She opened the door. The gas burned low. I lay on my right side, arms folded. She asked me if I needed anything. I muttered an incoherent sentence, the only word of which she could distinguish was "tobacco." She asked what I said. I muttered the same sentence. She retired to bed, but not to sleep. At 2.30 A. M., 23d ult., I awoke, surprised and vexed to find myself still alive. I was wet through with a cold perspiration; my pulse beat so slowly (unless I am mistaken) that I tired of trying to count it; my brain was perfectly clear; my legs were useless. I sat on the side of the bed leaning back on my elbow, fearing that I might pitch headlong on the floor and disturb the house. Remembering a forgotten message, I pitched along to my table and tried to write. My sight was dim. I could not control the arm, but after a fight of will wrote legibly. The door opened again, my back to it. The good lady asked, "Are you sick? Can I do anything for you?" to which I shook my head and she retired at ease in mind. I could not control the mouth muscles to articulate. Then, pouring out another horn of whisky and supplementing it by full *thirty* grains of morphine, I resumed the former position on the bed at 3.15 A. M. At 4.30 P. M. of that day the landlady rapped at my door. (It had been erroneously stated that A. M. that I went out early, so no one had been near.) "Do you need anything?"

*Since called and made a fuss, etc., of phial only chalk, etc.—no good. Biggest kind of oaths it came out of regular 8-phial oz. package, etc.

"No, thanks." "Will you be down to tea?" "Probably." I have no recollection of that dialogue, nor of the previous muttered sentence to the first lady. At 6 o'clock P. M. I awoke; had not moved a muscle. The same surprise and anger, the same cold perspiration. I felt and looked to see where I had vomited. Not a drop. No excretion from the body but sweat. I instantly swallowed the remaining fifteen grains as before, in desperation. Fell asleep exactly as before. At midnight I awoke and sat up, leaning against the wall, and expressed myself mentally thus: "Well, here's a hell of a go! Sixty grains of morphine in twenty-four hours and alive yet! Reckon my time has not come, my work not done yet. There may, after all, be 'a Divinity that shapes our ends rough.'" A tap at the door. I grunted. The lady entered, glanced at me, gave a scream, and fled. She had the sense to moderate her scream, ere it was all out, and returned. "Oh! how horrible you look! Are you raving mad? You look dreadfully! O horrors! what a sight!" and she put her hands before her eyes. My mouth and throat had not a drop of moisture, yet I was not in the least thirsty. I took hold of my chin and managed to articulate, "I'm sick, but all right." "What can I get you?" she asked. "All the cold coffee in the kitchen." She brought me a quart of the strongest and best of coffee. I drank it all. I asked her to make more. She did so, and in half an hour I drank another quart. I then asked for her husband. I felt that I wanted company. He came, a timid man. He was frightened at my looks, and I laughed to see him place his chair convenient to the open door so that he could "skip" in case I "tackled" him. I undressed and went to bed and slept a restless slumber. Since taking the first dose, a raw spot the size of a dime had appeared on my left temple, one on each knee, and one at the base of the spine. At 9 o'clock A. M., Sunday, 24th, a friend for whom I had sent arrived, also a Dr. R., allopath, a man of large practice, a grand man, and a friend of mine. When I told him what I had done he would not believe it. "I had vomited." "Show me where? Examine the vessel, every place; I have not left this room." "Ah! suspecting your motive, they gave you a harmless mixture." He took the phial and later tested the particles adhering to its bottom and sides. They stood the test as genuine. He stated the case to two contemporaries and they scorned the statement as impossible. I don't think he believes me to be a liar, but I'm sure he doubts my accuracy as to quantity. He is welcome. I *know* I took sixty grains in twenty-four hours. He only ordered more coffee and a prescription to open bowels—Podophyllin. No effect. Another of castor and croton oils. At midnight, a slight movement aided by injection. For several days was very weak and sleepless, and yet I'm not sure I was any weaker or more sleepless than before the doses. He gave me pills composed of strychnine, phosphorus, cayenne, and iron. As usual, they made me sick; as usual, I threw them away and improved. Am still in much the weak and useless state I was prior to February 22d. Said Dr. R. very earnestly: "You have a constitution of iron, a will of steel, and brains enough for two men. Don't try to back out again. You have a high mark to make in this world yet." If any one will kindly loan me a piece of chalk, I'll stand on a chair and make the mark as high on the wall as possible.

N. H., Ct., *March* 16*th*, 1884. J. S. H.

